# Stimulation of resistance genes and antioxidant enzymes in lettuce by nano metal oxides against root rot caused by *Rhizoctonia solani*

**DOI:** 10.1371/journal.pone.0334506

**Published:** 2025-10-14

**Authors:** Tarek A. Essa, Nashwa A. H. Fetyan, Tamer M. Salem, Nazih Y. Rebouh, Abdelaziz Kishk, Mohamed H. Abdelfattah

**Affiliations:** 1 Vegetable Diseases Research Department, Pant Pathology Research Institute, Agricultural Research Center, Giza, Egypt; 2 Agricultural Microbiology Research Department, Soils, Water and Environment Research Institute, Agriculture Research Center, Giza, Egypt; 3 Environment Research Department, Soils, Water and Environment Research Institute, Agriculture Research Center, Giza, Egypt; 4 Department of Environmental Management, Institute of Environmental Engineering, RUDN University, Moscow, Russia; 5 Department of Plant Protection, Faculty of Agriculture, Tanta University, Tanta, Egypt; 6 Genetic Department, Faculty of Agriculture, Tanta University, Tanta, Egypt; University of Agriculture Faisalabad, PAKISTAN

## Abstract

Root rot, caused by *Rhizoctonia solani* L., is becoming an increasing issue for lettuce. Nanoparticles (NPs) are emerging as a promising approach for managing biotic stress, offering advantages surpassing traditional control methods. This studyaimed to evaluate the effectiveness of silicon dioxide (SiO_2_), copper oxide (CuO), and gamma iron oxide (γFe_2_O_3_) nanoparticles in inducing systemic resistance (SR) in lettuce against *R. solani* by examining the molecular response, particularly the expression of pathogenesis-related and stress-regulatory genes. Additionally, assessed total protein contents, photosynthetic pigments, Hydrogen peroxide (H_2_O_2_), malondialdehyde (MDA), and antioxidant enzymes. The results demonstrated that NPs significantly reduced basal rot symptoms and decreased the Area Under the Disease Progress Curve (AUDPC) values. Treated plants also showed increased protein and chlorophyll levels compared to untreated controls. Infected plants showed higher levels of lipid peroxidation (MDA and H_2_O_2_). However, treatments with SiO_2_ and γFe_2_O_3_ effectively mitigated the oxidative stress. All NPs enhanced carotenoid content and antioxidant enzyme activity (superoxide dismutase [SOD], catalase [CAT], and ascorbate peroxidase [APX]), with γFe_2_O_3_ being the most effective. Importantly, NPs induced expression of *pathogenesis-related genes, PR1, PR3, and PR4*, as well as *the Ethylene-Responsive Transcription Factor 1A gene (ERT1)*. The upregulation of these genes was correlated with reduced disease symptoms and improved physiological status, indicating that enhanced gene expression contributed to the observed systemic resistance. This is the first study to report *the fatty acid hydroperoxide lyase (FHL) gene* activation in lettuce treated with CuO, γFe₂O₃, and SiO₂ nanoparticles against *R. solani*. These findings suggest that NPs are promising for managing *R. solani* in lettuce, applicable in both greenhouses and fields by enhancing systemic resistance.

## 1. Introduction

Agriculture is commonly regarded as the foundation of numerous countries across the globe. However, it faces many global challenges. For example, diseases caused by fungi and other infections that inhabit the environment lead to significant crop losses [1–4]. Among these pathogens, *Rhizoctonia solani*, a soil-borne plant pathogen characterized by a wide variation in culture morphology, host, and aggression and the most prominent species within the *Rhizoctonia* genus. *R. solani* causes severe diseases such as root rot, damping-off, stem canker, and leaf blight. Furthermore, it causes significant economic losses in many vital areas and horticulture crops worldwide [5]. In particular, *R. solani*-induced root rot is a widespread and destructive disease that severely impacts the growth and yield of lettuce (*Lactuca sativa* L.) at various developmental stages [6]. The pathogen’s ability to persist in diverse habitats, including plant debris and soil, and to infect a wide range of hosts makes its management especially difficult [7]. Moreover, the availability of lettuce cultivars with strong resistance to *R. solani* remains limited [8].

Fungicides are a common and effective chemical control method against *R. solani*-associated diseases [9]. Significant problems arise when fungicides are used excessively and continuously, including the development of resistant strains of pathogens, harmful to beneficial organisms, environmental impact, human health risks, and economic implications [10]. Consequently, it became necessary to develop sustainable, effective, and eco-friendly control strategies against this persistent pathogen.

Inducing systemic resistance in the host plant has become an essential target for reducing disease severity at a low cost and with minimal environmental impact [11,12]. In this context, microbial biocontrol agents and organic amendments provide eco-friendly and sustainable solutions for controlling root rot disease in lettuce. These agents improve soil quality and support the growth of beneficial microbial populations. Additionally, plant-based compounds serve as a natural substitute for chemical fungicides, although their efficacy may need further refinement [13]..

Nanoparticles (NPs) are considered a potential strategy for reducing the damage wrought by abiotic and biotic stress. NPs serve as novel elicitors for the induction of plant resistance and enhance the antibacterial activity against plant diseases. Additionally, NPs represent a cutting-edge approach to improving crop production, quality, and protection [14,15]. Metal oxide NPs are crucial as a biostimulant to support the development and growth of crops under diverse stresses by penetrating the plants through roots or leaves, entering the cells, and causing modifications including stimulating secondary metabolites, activating antioxidant defenses, and influencing gene expression linked to stress tolerance [16]. For instance, iron oxide nanoparticles (Fe₂O₃ NPs)_,_ have proven effective in mitigating the detrimental effects of *Fusarium oxysporum* on infected eggplantswhile enhancing antioxidant enzymes activity, osmolyte accumulation, and photosynthetic pigment levels [17].

Previous studies have reported that the use of nano-biotechnology in pathogen resistance and the stimulation of plant physiological immunity have shown significant and promising results in combating fungal diseases. This approach enhances systemic immunity, strengthens disease resistance, and improves plant productivity [18–20]. As well, Zinc oxide (ZnO NPs) and Fe₂O₃ NPs have also proven effective in controlling *Fusarium* wilt disease by enhancing and promoting the production of biochemical compounds responsible for the defense of tomato plants [21,22].

Many previous studies have indicated the role of nanotechnology in stimulating the plant defense system against many pathogenic fungi by improving and developing biochemical compounds responsible for the defense and activity of antioxidant enzymes, osmolytes, and photosynthetic pigments. However, the role of nanotechnology in stimulating the genes responsible for resistance needs further study, especially in lettuce plants. Herein, we hypothesize that the application of silicon dioxide (SiO_2_), copper oxide (CuO), and gamma iron oxide (γFe_2_O_3_) nanoparticles possesses direct antifungal action against *R. solani*. As well, these NPs are expected to stimulate the systemic defense against root rot infection in lettuce seedlings in the greenhouse. In this study, we used quantitative real-time PCR (qRT-PCR) to determine the expression of defense and regulatory genes that induce resistance and to investigate the effects of metal oxide nanoparticles on some physiological characteristics of lettuce plants. We believe that the application of metal oxide nanoparticles against *R. solani* potentially provides a promising tool for effectively managing root rot disease in lettuce.

## 2. Materials and methods

### 2.1. Chemicals and reagents

Deionized water produced by a Milli-Q Plus with a resistivity of 18.2 MΩ cm was applied for sample preparation and standards. Acetone ≥99.5% (179124), Trichloroacetic acid ≥99.0% (76-03-9), Thiobarbituric acid 98% (504-17-6), and potato dextrose agar medium were purchased from Sigma Aldrich (Saint Louis, MO, USA). Tolclofos-methyl (fungicide), all the chemicals were used directly without further purification as it was of analytical grade.

### 2.2. Production of nanomaterial

The Nano metal oxides used in this study were previously synthesized and characterized in our previous research [23]. In brief, nanosilica was synthesized from paddy husk ash using the procedure detailed by Premaratne et al. [24]. Magnetic nanoparticles (MNPs) were also synthesized by the method described in Jeon et al. [25]. Nano-copper was produced through a chemical precipitation process involving the reduction of copper salt in an aqueous solution [26,27]. The surface morphology of the synthesized materials was investigated by a scanning electron microscope (SEM) (H600 Electron Microscope, Japan) as documented previously [23].

### 2.3. Isolation and identification of the Fungal Pathogen

Pathogenic isolates of *R. solani* previously identified in our previous research [23] were utilized. In brief, roots and bottom stems were cleaned, treated with 3% hypochlorite solution for 3 min, and cultured on a potato dextrose agar (PDA) medium to promote fungal growth. The isolated fungus was identified according to the method described previously [28,29]. Furthermore, a mixture of rice grains, sand, and water was sterilized to create a rice hull medium. Fungal mycelium was then added to the mixture, and it was cultured for one week at 28° C [23].

### 2.4. Assessment of the response of lettuce root rot to nanoparticle oxide materials in a greenhouse environment

**Fig 1:**
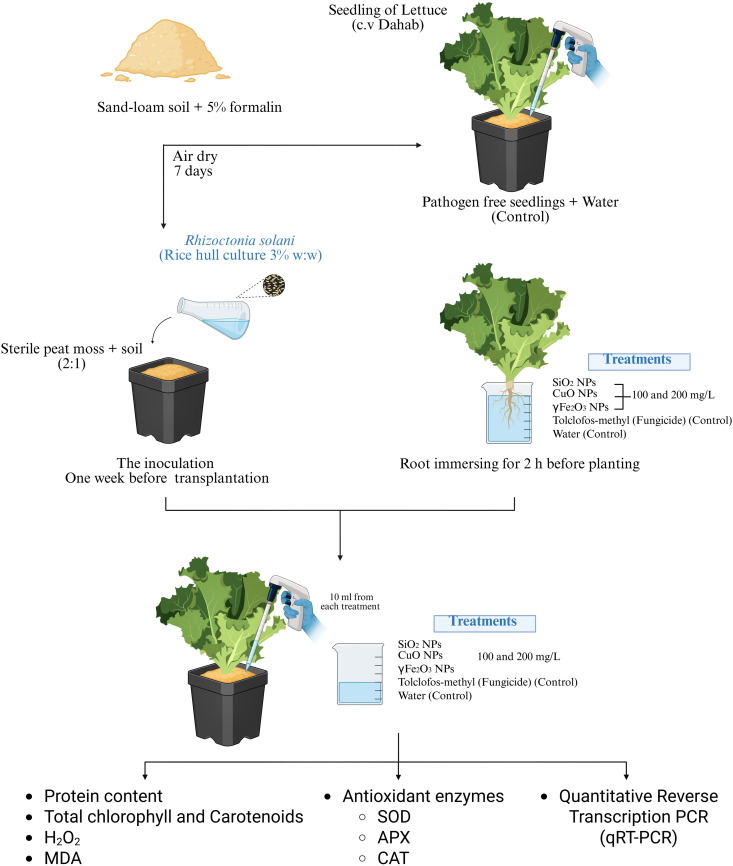
The experimental design designed to quantify numerous Nano particles (NPs) effects on total protein contents, Total chlorophyll and carotenoids, Hydrogen peroxide (H_2_O_2_), malondialdehyde (MDA), and antioxidant enzymes (Superoxide dismutase SOD, catalase CAT, and APX) of lettuce (*Lactuca sativa* L.) seedlings, in comparison to different controls.

The antifungal efficacy of metal oxide nanoparticles in the pot experiment was assessed on the second week of September in 2024 under greenhouse conditions at Agricultural Research Station, Agricultural Research Center (ARC), Egypt; Maine (Latitude 30°8’22“N, Longitude 31°15’50”E, “) in Giza Governorate, Egypt. The soil texture was a sandy clay–loam soil with the following characteristics: sand 45.5%, silt 20.0%, loam 34.5%, pH 7.6, EC 1.36 ds/m, Organic matter 1.85%, total nitrogen 0.13%, total phosphorus 0.025%, and available phosphorus 0.005%. To assess the fungal activity, sand-loam soil has been sterilized by 5% formalin and allowed to air dry for seven days [30]. Each pot, 25 cm in diameter, was filled with a sterile substrate mix of 2:1 peat moss and sterilized soil. One week before transplantation, the soil was infected with *R. solani* by a rice hull medium (3% w/w). Lettuce seedlings cv. Dahab (obtained from Vegetables Research Department, Research Institute, Agricultural Research Center, Giza 12619, Egypt). Thirty-day-old seedlings (from sowing) were treated with suspensions of silicon dioxide (SiO₂), copper oxide (CuO), and gamma iron oxide (γ-Fe₂O₃) at concentrations of 100 and 200 mg L ⁻ ¹ for 2 hours prior to planting; These concentrations were chosen because they were the most effective among the three tested levels (50, 100, and 200 mg L ⁻ ¹). Our previous research showed that 100 and 200 mg L ⁻ ¹ significantly inhibited fungal growth without causing phytotoxic effects when the same nanomaterials were applied to the same pathogen and crop [23]. The same solutions were used to drench the soil around the lettuce roots, with 10 mL injections. The experiment followed a completely randomized design (CRD), with each treatment replicated three times, and each replicate consisting of three lettuce plants. Distilled water was used as the control. **Fig 1** displays the experimental design, which compares the effect of various nanoparticles on infected lettuce seedlings to controls. The plants were grown to maturity (55 days post-transplanting). The irrigation was conducted twice per week (200 ml/ pot), the pots were fertilized with a balanced (N: P: K) 20:20:20 every week at a dose of 1g/L, as well as minor elements (Fe, Mn, Zn, …etc.) applied directly to the root zone. Fertilization was continued throughout the growing season based on plant developmental and visual assessment of nutrient status. The greenhouse temperature was 23 ± 2 °C during the trial. Tolclofos-methyl (Fungicide: Organic Thiophosphate) (Dongying Shengya Chemical Co., Ltd., Dongying, Shandong, China) (2.5 g/L) served as a positive control.

Afterward, the severity and incidence of the disease were recorded four times at 7-day intervals, beginning 20 days after transplantation., and computed according to Liu et al. [31] and Abd-El-Khair and El-Nagdi [32] based on the following equations.


$$Diseaseincidence(\%)=\frac{Numberofinfectedplants}{Totalnumberofplants}\text{X 100}$$

$$Diseaseseverity(\%)=\frac{\Sigma n\text{x}r}{\text{5}N}\text{X 100}$$

While n represents the plant numbers at each numerical rate, N is the total number of plants multiplied by the highest numerical rate, and r is equal to 5.

In brief, disease severity was assessed by monitoring the progression of yellowing, root rot, and overall plant decay at the end of the experiment. The assessment of disease severity was conducted using a modified 0–5 scale, where 0 = 0% (no symptoms), 1 = 0–10%, 2 = 10–25%, 3 = 25–50%, 4 = 50–75%, and 5 = 75–100% (severe symptoms or plant death).

Additionally, the severity of the disease was evaluated using a four-category assessment [33]. Based on the information at hand, the area under the disease progression curve (AUDPC) was calculated using the following formula to compare the different responses of the plants under study [34]:


$$\text{AUDPC}=\text{D}[\text{1}/\text{2}({\text{Y}}_{\text{1}}+{\text{Y}}_{\text{K}})+{\text{Y}}_{\text{2}}+{\text{Y}}_{\text{3}}+\ldots .+{\text{Y}}_{\left(\text{K}-\text{1}\right)}]$$

Where:

Y1 is the first injury record, and D is the number of days between each subsequent two measurements. Last injury record, Yk.

### 2.5. Estimation of total protein contents

About 1.0 ml of cold extraction buffer was used to homogenize around 0.5 g of lettuce leaf that had been ground with liquid nitrogen [35]. The Bradford approach was used to determine the quantity of the isolated proteins [36]. Bovine serum albumin (Sigma) was used to create the protein standard curve at different concentrations (10−100 μg ml^-1^). All measurements were performed in three biological replicates.

### 2.6. Estimation of photosynthetic pigments

Approximately 0.5 g of lettuce leaves were immersed in five mL of acetone/water (80% v/v) and kept in the dark at 5 °C for 48 hours, wrapped in aluminum foil to prevent pigment degradation by light to measure total chlorophyll by measuring both chlorophyll-a and chlorophyll-b in three biological replicates. Also, carotenoids were measured [37]. The used equations are as follows:

$\text{Chlorophyll a}(\text{mg}/\text{mL}=\text{0}.\text{0127}\times A_{\text{663}}–\text{0}.\text{0027}\times A_{\text{645}}$

$\text{Chlorophyll b}(\text{mg}/\text{mL}=\text{0}.\text{0229}\times A_{\text{645}}–\text{0}.\text{0046}\times A_{\text{663}}$

Total chlorophyll(mg/mL=Chlorophyll a+Chlorophyll b

$\text{Carotenoid}(\text{mg}/\text{mL}=A\text{470}+\text{0}.\text{1140}\times A_{\text{663}}–\text{0}.\text{6380}\times A_{\text{645}}$

### 2.7. Hydrogen peroxide (H_2_O_2_) determination

After homogenizing 100 mg of fresh leaf samples with one mL of 0.1% (w/v) trichloroacetic acid, the samples were centrifuged for 15 minutes at 10,000 rpm at room temperature. One millilitre of supernatant was combined with one millilitre of 0.01 M phosphate buffer (pH 7) and two millilitres of a potassium iodide solution (1 M) to measure the H_2_O_2_ concentration. H_2_O_2_ concentration was measured at A_390_ by a spectrophotometer (UV1901PC) against a different standard solutions of H_2_O_2_ at various concentrations (from 0.1 to 1 mM) [38].

### 2.8. Lipid peroxidation

Lipid peroxidation was determined by measuring the amount of thiobarbituric acid reactive substances that were equal in concentration to malondialdehyde (MDA) [39].

### 2.9. Antioxidant enzymes assay

About one gram of frozen leaves was homogenized in 0.05 M sodium phosphate buffer solution (pH 7 for Catalase and pH 7.8 for Superoxide Dismutase). Then, a frozen leaf was homogenized in five ml of 0.05 M Tris-HCl buffer for ascorbate peroxidase. The mixtures were centrifuged at 12,000 × g for 10 minutes at 4 °C. The supernatant was utilized to determine enzyme activity. Superoxide Dismutase (SOD) activities were evaluated based on the enzyme’s ability to inhibit the photochemical reduction of nitroblue tetrazolium (NBT) [40]. Meanwhile, Catalase (CAT) activity was measured by monitoring the decrease in H₂O₂ absorbance at 240 nm, where the rate of reduction reflects enzyme activity [41]. Finally, the APX activity was assessed using Nakano and Asada [42] procedure.

### 2.10. RNA extraction and complementary DNA synthesis

Total RNA was isolated from leaves, which were stored at −80 °C for seedlings under control and other treatments using an RNA extraction kit *(***Thermo Scientific, Fermentas, #BSC52****S1***).* The amount and purity of extracted RNA were measured using the Nanodrop (**Analytikjena SCANDROP 200/Germany**). To synthesize first-strand cDNA, one μg of total RNA, with a purity of more than 1.8, was added to a 20 μL reaction mix using a procedure supported by reverse transcription kits (**Thermo Scientific, Fermentas, #EP0451**).

The quantitative measurement of gene expression was done using SYBR Green low Rox stain (Thermo Scientific, USA, # BSC52S1). The reaction was performed in a reaction volume of 20 μL. The final reaction mixture was placed in a real-time quantitative PCR thermal cycler (Qiagen Rotor-Gene Q 5plex HRM, Germany). *Actin* gene (Ac: JX444700.1) was used as an endogenous control. To adjust the annealing temperature for various genes on the same plate, the reaction was conducted using the Variflex tool. Gene-specific primers were designed based on the lettuce gene sequence available on the NCBI website (https://www.ncbi.nlm.nih.gov). The accession number for the gene sequence has been provided in Table 1. Each sample was tested in 3 biological replicates, with each reaction run in triplicate (technical replicates) using the same gene-specific primers. The qRT-PCR amplification conditions were as follows: 95 °C for 5 min; 40 cycles at 95 °C for 20 s; 56–61°C for 30 s according to annealing temperatures for each primer; and 72 °C for 45 s. A melting curve analysis was performed by gradually heating the amplicon from 55 °C to 95 °C to confirm primer specificity.. Relative expression (RQ) was calculated as 2^-∆∆ct^ calibrated with the reference gene and control treatment [43].

**Table 1:** List of primers used in quantitative RT-PCR.

Gene code	Function	Ac. number	Sequence (5- to 3-)
Actin	Used as an endogenous control	(JX444700.1)	F: TGGAGATGATGCACCTCGTG R:CACGCTTAGACTGTGCCTCA
PR-1	pathogenesis-related protein PR-1	XM_023876054.3	F: TCCAGAACAAAGCCCGATCT R: CCGCTTCCCCAAAAGATGTT
PR-3	pathogenesis-related thaumatin-like protein 3.5	XM_023914858.3	F: GTACAACGCTAACCTGCACC R: CACTCCGACATGCAACTGTT
PR-4	pathogenesis-related leaf protein 4-like	XM_052765945.1	F: TACTCACAATGCAGCTCGTG R: TGTCCACCAGCACAAGTATT
ERT1	ethylene-responsive transcription factor 1A	XM_023900790.3	F: ATAGGATGCGTGGTTCTCGT R: CTGCCACGTAGATCTGTTGC
FHL	fatty acid hydroperoxide lyase	XM_023907774.1	F: GGAAAACTCACCGGACATGG R: CGTCTTTGCCCTCTTTCTCG

Variations on the expression patterns of some genes; *pathogenesis-related (PR) proteins*, such as *PR1* proteins (antifungal), *PR3* (chitinases), *PR4* proteins (antifungal) and *Ethylene-Responsive Transcription factor 1A gene (ERT1)* as a key regulatory in plant reaction to biotic stresses *and the fatty acid hydroperoxide lyase gene (FHL)* were detected using quantitative real-time PCR analysis. *Actin* gene (Ac: JX444700.1) was used as the internal reference gene for calibration of the expression level of studied genes.

### 2.11. Determination of Si, Cu, and Fe concentrations in plants

Six plants from each treatment and the controls were chosen randomly after 45 days of transplantation and collected to determine the residues of Si, Cu, and Fe contents and to evaluate the absence of the identified nanoparticle using single particle Inductively Coupled Plasma Mass Spectrometry (spICP-MS). The plants were dried and converted to ashes at 500 °C in a muffle furnace (Furnace Type 6000, Tourmaline). The ash was wet digested using a mixture in a 7:3 ratio. The concentrations of Si, Cu, and Fe were determined by Inductively-Coupled Plasma Optical Emission Spectroscopy (ICP-OES) [44]. Total concentrations were tested using recognized reference material (GBW 10015 Spinach, Institute of Geophysical and Geochemical Exploration, Langfang, China). For spICP-MS, particle size calibration used NIST-certified 60 nm silver standards, and transport efficiency was calculated by the frequency method. ICP-OES was calibrated with certified multi-element standards using matrix-matched solutions. Accuracy was confirmed with GBW 10015 (Spinach) reference material.

### 2.12. Statistical analysis

Analysis of variance (ANOVA) and post-hoc Tukey’s test were used to examine all of the data, with a significance threshold of 0.05. Ward’s minimal variance was used as the basis for a two-way hierarchical cluster analysis (HCA), Error bars in graphs represent the standard error of the mean (SE). Furthermore, the purpose of the heat map was to show multivariate commonalities between treatments [45]. The dataset’s dimensionality was decreased, and underlying treatment patterns were found using principal component analysis (PCA). To run ANOVA, Tukey’s tests, HCA, heat map, and PCA, JMP Data Analysis Software Version 15 (SAS Institute Inc., 270 Cary, NC, USA) was utilized.

## 3. Results and discussion

### 3.1. The Relationship between *R. solani* and nanoparticle oxides on disease Development

**Fig 2:**
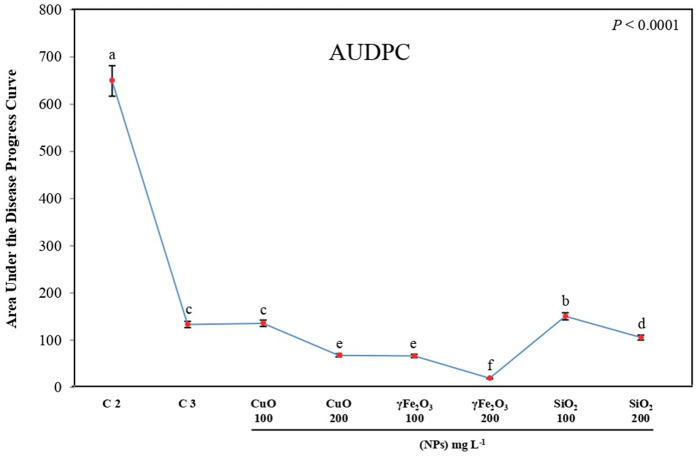
Impact of different nanoparticles, CuO, γFe_2_O_3_, and SiO_2_ at two different concentrations (100 and 200 mg L-1) on *L. sativa* infected seedlings compared to the controls (C1: healthy plants, C2: infected plants, C3: infected plants + Tolclofos-methyl) on the area under the disease progress curve (AUDPC).

In this trial, increasing the dose of nanoparticle oxides resulted in a decrease in the area under the disease progress curve (AUDPC). Lettuce plants grown in infected soil displayed severe basal rot symptoms and had a high AUDPC value. All nanoparticle oxides used in this study effectively reduced *R. solani* basal rot symptoms and yielded low AUDPC values. Treatment with γFe_2_O_3_ nanoparticles at 100 and 200 mg L^-1^ produced the lowest values, 66.75 and 19.25, respectively, compared to treatments with other nanoparticle oxides. Treatments with CuO and SiO_2_ nanoparticles at 200 mg L^-1^ were the best effective in the reduction of *R. solani* basal rot symptoms in lettuce plants than treatment with Tolclofos-methyl fungicide (Fig 2).

The obtained results suggest that a severe infection of *R. solani* produces root rot in lettuce plants in the inoculated treatment. Application of SiO_2_, CuO, or γFe_2_O_3_ nanoparticles using dipping and soil drench treatments was the most effective technique for reducing *Rhizoctonia* root rot. Nanoparticles can disrupt microbial cell membranes, leading to leakage of cellular contents and inhibition of pathogen growth [46]. Our findings are consistent with previous studies indicating that metallic nanoparticles, including silver, copper, and zinc oxide, as well as organic nanoparticles and composite formulations, exhibit potent antimicrobial properties by effectively inhibiting pathogen proliferation and disrupting infection-related processes [47]. Dimkpa et al. [48] reported that copper oxide (CuO) and zinc oxide (ZnO) nanoparticles mitigate Fusarium wilt in tomato (Solanum lycopersicum) while concurrently enhancing plant growth. The dual functionality is attributed to the nanoparticles’ antifungal activity against Fusarium oxysporum and their role in stimulating host defense responses, potentially through improved nutrient acquisition and regulation of defense- and growth-related gene expression. Fe_2_O_3_ NPs may affect the pathogen by inducing greater antioxidant enzyme activity, which is related to iron’s role in RNA synthesis and enzyme activity. SiO_2_ NP treatment demonstrated significant potential, probably due to the creation of physical barriers produced by silicon buildup within plant cells [49]. CuO Nanoparticles interact with pathogens by penetrating the membranes of cells, oxidizing lipids of membranes, denaturing nucleic acids, and altering proteins, which ultimately leads to cell death [50]. AgNPs have demonstrated broad-spectrum antimicrobial activity against plant pathogens such as Alternaria solani in tomatoes and Xanthomonas oryzae in rice. These nanoparticles inhibit disease progression by activating antioxidant enzymes and modulating reactive oxygen species (ROS) homeostasis [51]. ZnO NPs have been effective in controlling Fusarium wilt in common beans, with disease control rates reaching up to 82.77% under protective treatments [52].

### 3.2. Total protein contents

**Fig 3:**
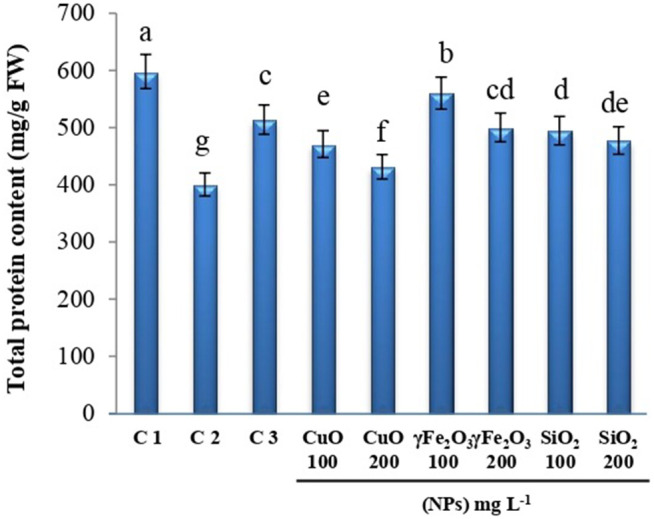
Changes in total protein content of lettuce leaves under all experimental conditions. C1: healthy plants, C2: infected plants, C3: infected plants + Tolclofos-methyl (fungicide) and the infected plants with three nanoparticles, CuO, γFe_2_O_3_, and SiO_2_ at two different concentrations (100 and 200 mg L^-1^). Data are shown as mean ± standard error of the mean (SE). Values with different letters are significantly different at *p* ≤ 0.05 as analyzed using One-way ANOVA followed by Tukey’s test.

Quantification of total protein contents in lettuce leaves showed a significant decrease in infected plants with *R. Solani* in comparison with non-infected plants, while plants treated with fungicides or Nano metal oxides showed a significant increase in protein contents compared to non-treated plants (**Fig 3**). Treated plants with Nano metal oxides showed that; the highest value of protein contents (0.56 mg g^-1^ FW) was obtained in treatment with 100 mg L^-1^ γFe_2_O_3_ where it increased significantly compared with non-treated plants. Overall, total protein levels were considerably lower in plants treated with greater concentrations (200 mg L^-1^) of all evaluated nanoparticles compared to 100 mg L^-1^.

Soil fungi can occasionally affect lettuce, causing damping-off. The seedlings can be killed before or soon after emergence. In addition, infected seedlings exhibit decaying roots and dark lesions on their stems. Subsequently, the stem tissue of infected plants collapses, causing the plants to fall over and die [7]. Usually, diseased plants show reductions in protein content; Similar conclusions were observed by Akkurak et al [53]. Infected lettuce plants had considerably decreased soluble protein content following phytoplasma infection. As well, protein contents in seedlings of pepper plants infected with soil fungus were reduced severely (about 68.17%) [54]. In this study, data showed that plants treated with CuO, SiO_2_, and γFe_2_O_3,_ especially at 100 mg L^-1^ nanoparticles, demonstrated a significant increase in total proteins relative to untreated plants. Similarly, applying CuO NPs (100 ppm) to cowpea plants reduced the severity of root-knot disease while improving growth, production, and the level of protein [55]. Where reduction in protein content at high concentrations of nanoparticles especially CuO nanoparticles may be due to the toxicity of plants at this concentration. In the same manner, plants treated with CuO nanoparticles exhibited enhanced toxicity in terms of plant biomass decline [56], similar to previous results of cucumber plants treated with CuO nanoparticles grown in soil or hydroponically [57]. Previous studies reported that Fe_3_O_4_ nanoparticles increased plant growth parameters and enhanced nitrogen, phosphorus, and potassium uptake, which stimulated the growth of plants [58]. This aligns with our results, which showed that γFe_2_O_3_ treatment increased protein content relative to other treatments. In addition to disease control, certain nanoparticles have been reported to promote plant growth. Foliar application of ZnO NPs at 10 mg/L has been shown to enhance growth parameters such as stem length, root volume, and nutrient uptake in mung bean plants. Application of Fe NPs at 25 mg/kg has improved growth in wheat, particularly under salt-affected conditions, by enhancing dry matter accumulation and nutrient uptake. These findings align with our observations, indicating that γFe_2_O_3_ not only control pathogens but also promote plant growth [59].

### 3.3. Changes in photosynthetic pigments

**Fig 4:**
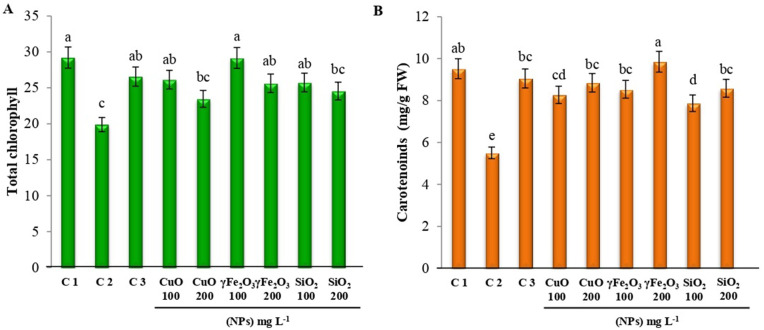
Changes in (A) total chlorophyll and (B) carotenoids of lettuce leaves after treatment using three different nanoparticles, CuO, γFe_2_O_3_, and SiO_2_ at two different concentrations (100 and 200 mg L^-1^) on *L*. *sativa* infected seedlings. Healthy plants (C1), infected plants C2, and infected plants + Tolclofos-methyl (C3) were used as controls. Data are shown as mean ± standard error of the mean (SE). Values with different letters are significantly different at *p* ≤ 0.05 as analyzed using One-way ANOVA followed by Tukey’s test.

Changes in the amounts of total chlorophyll and carotenoids in plants under all studied treatments are shown in **Fig 4A** and B. Infected plants that received no treatments showed the lowest total chlorophyll levels. While plants treated with fungicide or nano metal oxide displayed a considerable increase in total chlorophyll in comparison to non-treated plants (**Fig 4A**). Treated plants with γFe_2_O_3_ at 100 mg L^-1^ showed the highest value of total chlorophyll. Also, similar to total protein the high concentration of Nano metal oxide (200 mg L^-1^) reduced total chlorophyll and CuO showed the lowest value compared to other Nano metal oxide. Infected plants exhibited much lower carotenoid content than control plants, whereas treated plants had significantly higher carotenoid content than non-treated plants. The highest carotenoid content was recorded with the γFe_2_O_3_ at 200 mg L^-1^ (Fig 4B). In contrast to chlorophyll, high concentrations of nano (200 mg L^-1^) showed an increase in carotenoid content. The main function of these pigments (chlorophyll and carotenoids) in the photosynthesis process is the absorption of light to excite the electrons in the pigments [60]. *A. solani* causes a significant decline in photosynthetic pigments subsequently, growth parameters. Furthermore, leads to significant declines in the quantities of carbohydrates and protein [61]. Numerous research has confirmed enhanced development and production in plants treated with Fe_3_O_4_ nanoparticles, which also improved total chlorophyll, carotenoids, proteins, and total amino acid contents in plants [58]. The results revealed that the exogenous application of Si nanoparticles promoted the growth and boosted photosynthetic characteristics including carotenoids and total chlorophyll, in *Coleus aromaticus* [62]. CuOnanoparticles (100 ppm) were applied to cowpea plants before the Meloidogyne incognita inoculation, which increased growth, yield, carotenoids, and chlorophyll levels while lessening the severity of root-knot disease [55]*.* Current research suggests that certain nanoparticles can enhance the activity of enzymes known as antioxidants in plants, therefore protecting chlorophyll and plant cells and decreasing ROS-induced oxidative stress [63]. Furthermore, ROS-like singlet oxygen can directly or via secondary radicals assault and degrade photosynthetic apparatus component proteins, including the D1 protein of photosystem II (PSII) [64]. Carotenoids are recognized as light-catching pigments that capture photons and transfer the excitation energy to chlorophyll. In addition, they are effective quenchers of ROS, notably singlet oxygen (^1^O_2_) [65].

### 3.4. Oxidation of lipids and hydrogen peroxide

**Fig 5:**
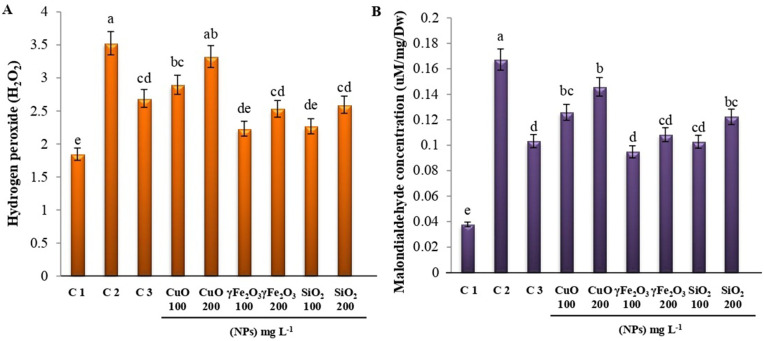
Changes in (A) H_2_O_2_ and (B) MDA contents of lettuce leaves after treatment using three different nanoparticles, CuO, γFe_2_O_3_, and SiO_2_ at two different concentrations (100 and 200 mg L^-1^) on *L*. *sativa* infected seedlings. Healthy plants (C1), infected plants C2, and infected plants + Tolclofos-methyl (C3) were used as controls. Data are shown as mean ± standard error of the mean (SE). Values with different letters are significantly different at *p* ≤ 0.05 as analyzed using One-way ANOVA followed by Tukey’s test.

Oxidative stress was indicated by changes in H_2_O_2_ and lipid peroxidation products (MDA) contents across all experimental treatments. The data presented in **Fig 5** showed a considerable increase in H_2_O_2_ (**Fig 5A**) and MDA (**Fig 5B**) contents in infected plants compared to uninfected plants. Treated plants with fungicide or Nano metal oxide reduced H_2_O_2_ and MDA contents in comparison with non-treated plants. The lowest levels of MDA and H_2_O_2_ contents were recorded in plants treated with γFe_2_O_3_ at 100 mg L^-1^ and SiO_2_ at 100 mg L^-1^, while plants treated with CuO at 200 mg L^-1^ showed a significant increase in MDA and H_2_O_2_ contents. Generally, as the level of nanoparticles increased from 100 to 200 mg L^-1^, the amount of H_2_O_2_ and MDA produced was significantly increased. The early plant reaction upon pathogen detection is the hypersensitive response, which results in the generation of ROS, predominantly superoxide (O^-2^) and H_2_O_2_, at the location of attempted invasion [66]. High ROS production is hazardous for plants because it causes damage from oxidative stress, induces apoptosis and necrosis, and eventually leads to plant cell death. Low levels of ROS, on the other hand, have been shown to behave as signaling molecules in the development of plants and stress reactions [67]. Additionally, low or moderate NP doses inhibited ROS production in plants [68]. Plants use their antioxidant defenses to counteract the ROS generated by NP interaction, allowing them to gradually adapt to stress situations [69,70].

### 3.5. Antioxidant enzyme activity

**Fig 6:**
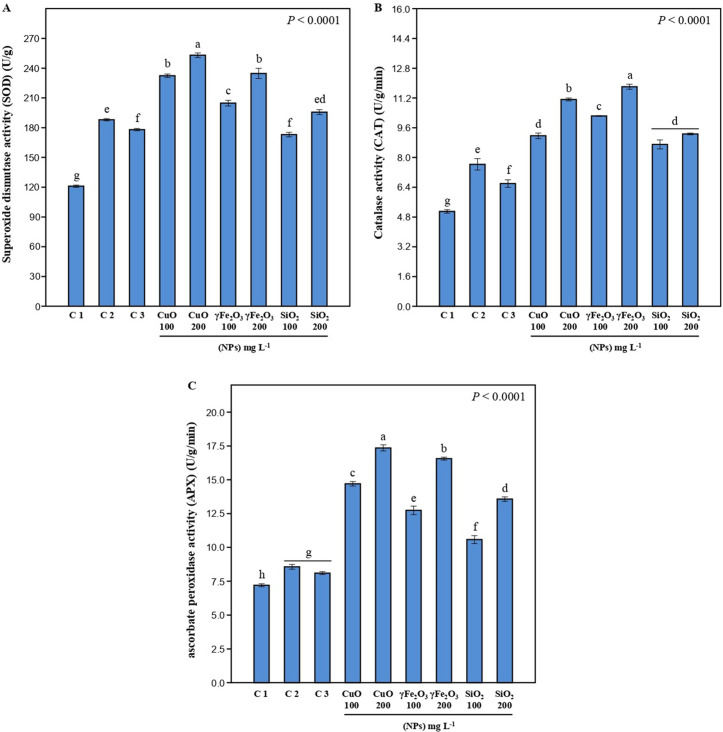
Activity of (A) Superoxide dismutase [SOD], (B) Catalase [CAT], and (C) Ascorbate peroxidase [APX] of lettuce leaves after treatment using three different nanoparticles, CuO, γFe_2_O_3_, and SiO_2_ at two different concentrations (100 and 200 mg L^-1^) on *L*. *sativa* infected seedlings. Healthy plants (C1), infected plants C2, and infected plants + Tolclofos-methyl (C3) were used as controls. Data are shown as mean ± standard error of the mean (SE). Values with different letters are significantly different at *p* ≤ 0.05 as analyzed using One-way ANOVA followed by Tukey’s test.

The superoxide dismutase enzyme (SOD) acts as the plant’s first line of defense against oxidative stress by scavenging the superoxide anion and converting it into oxygen and hydrogen peroxide. Data presented in **Fig 6A** showed that SOD activity increased considerably in infected plants in comparison to uninfected plants. Both CuO and γFe_2_O_3_ treatments significantly enhanced SOD activity relative to untreated plants. The obtained results showed that the activity of SOD increased with higher concentrations of nano metal oxides. Similarly, CuO nanoparticles were reported to significantly increase SOD gene expression in *Cucumis sativus* [56]. Catalase is among the most significant antioxidant enzymes, which decomposes H_2_O_2_ into harmless compounds like water and oxygen, and is used for managing a variety of oxidative stress-related diseases. The fungal Stress infection in this study caused a notable rise in CAT activity compared to uninfected plants (**Fig 6B**). All utilized Nano metal oxides showed a considerable increase in CAT activity in comparison with untreated plants. The highest CAT activity was recorded with γFe_2_O_3_ at 200 mg L^-1^ treatment (**Fig 6B**).

In plants, Ascorbate peroxidase (APX) is an essential antioxidant enzyme. APX enzyme produces the formation of oxygen and water from H_2_O_2_ to save plants from damage caused by high H_2_O_2_ accumulation. The activity of APX enzymes in infected plants was significantly higher compared to uninfected plants (**Fig 6C**). Treatment with CuO at 200 mg L^-1^ recorded the highest APX activity, followed by γFe_2_O_3_ at 200 mg L^-1^ treatment. The effects of SiO_2_ were less pronounced in inducing enzyme activity compared to CuO and γFe_2_O_3_. Many enzymes use iron as a cofactor involved in biochemical processes in plants. Recent studies confirmed that Fe_2_O_3_ nanoparticles induce changes in the activities of antioxidant enzymes in wheat and peanuts under stress conditions [71,72]. Foliar application of Fe_3_O_4_ nanoparticles significantly increased peroxidase (POX), polyphenol oxidase (PPO), superoxide dismutase (SOD), and nitrate reductase (NR) in *Moringa oleifera* [58]. The enhancement in CAT activity observed in lettuce leaves treated with CuO NP indicates that plant defense mechanisms are being activated against oxidative stress [73]. CuO nanoparticles applied via foliar spraying also induced a significantly increased production of bioactive substances as well as the activity of antioxidant enzymes like CAT and SOD in lettuce plants [74]. Plant treatment with SiO_2_ nanoparticles induced antioxidant enzyme activities in wheat plants under copper stress conditions [75]. The nanoparticles are involved in plant resistance enhancement by improving the ROS detoxification, which is triggered during fungal infection and thus maintaining the ROS homeostasis under pathogen attack [76]. In the present study, treatment with nanoparticles resulted in a significant enhancement of antioxidant enzyme activity. This elevated enzymatic response appears to be associated with a concomitant reduction in disease severity, suggesting a potential protective role of nanoparticles in mitigating oxidative stress-related pathological processes.

### 3.6. Principal component analysis and heat map illustrate the impact of nanoparticles on root rot in lettuce seedlings

**Fig 7:**
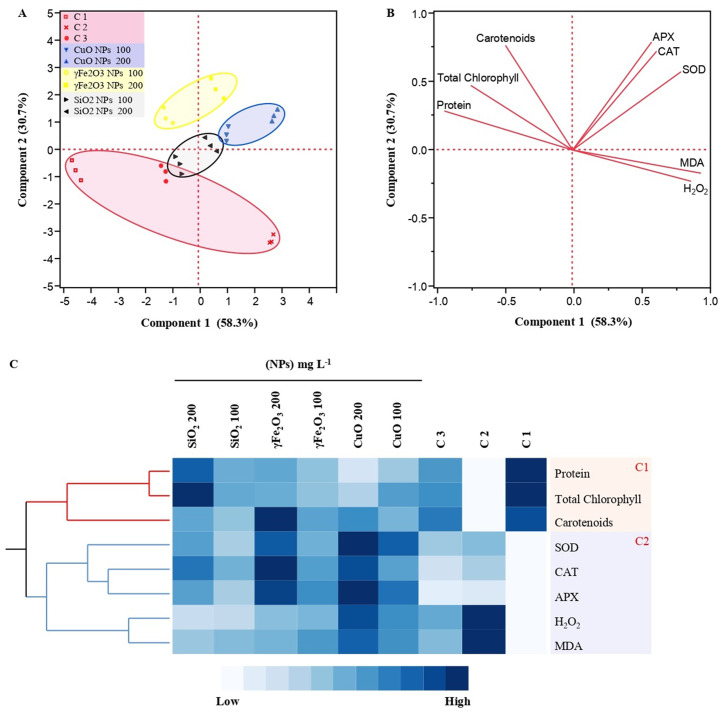
Principal component analysis (PCA) shows the multivariate variation among some physiological parameters and antioxidant enzyme activity after treatments with CuO, γFe_2_O_3_ and SiO_2_ nanoparticles at different concentrations (100 and 200 mg L^-1^) in comparison to the controls (C1: healthy plants, C2: infected plants, C3: infected plants + Tolclofos-methyl) on lettuce (*Lactuca sativa* L.) seedlings. (A) PCA-scatter blots. Colored symbols correspond to the total protein contents, total chlorophyll, and carotenoids, Hydrogen peroxide (H_2_O_2_), malondialdehyde (MDA), superoxide dismutase (SOD, catalase (CAT), and ascorbate peroxidase (APX) after the different treatments at different concentrations. (B) PCA-loading plot. The vectors shown in the figure indicate the direction and strength of each parameter. The two principal axes explain the variance. (C) A Two-way hierarchical cluster analysis and heat map illustrating the physiological parameters and antioxidant enzyme activity from all NP treatments and controls. The similar responses to the nanoparticles were categorized into groups in clusters 1 and 2. The cells represent the mean of the concentration of each parameter (n = 3).

The principal component analysis (PCA) biplot (**Fig 7A**) and loading plot (**Fig 7B**) together provide a comprehensive understanding of the impact of nanoparticles on physiological parameters and antioxidant enzyme activity in *L. sativa* L. seedlings. Together, PC1 and PC2 accounted for 89% of the total variance in the data, where PC1 contributed 58.3% and PC2 added 30.7% to the variance independently.

The PCA biplot indicated the distribution of treatments based on their principal component scores. Clusters of the controls (healthy plants, infected plants, infected plants + fungicide) were closely together, suggesting similar responses. Additionally, a significant impact of CuO nanoparticles on the parameters based on a distinct cluster separate from controls was detected. Moreover, γFe_2_O_3_ nanoparticles showed a distinct impact in comparison to both the control and CuO groups. Also, SiO_2_ nanoparticles showed a different impact compared to CuO and γFe_2_O_3_ nanoparticles (**Fig 7A**).

In the same manner, the loading plot direction and length presented the contribution of each parameter to the two principal components. Protein content, total chlorophyll, and carotenoids revealed high similarity as positioned close together. Likewise, SOD, CAT, and APX, clustered closely, responded similarly across different treatments. As well, H₂O₂ and MDA were located oppositely to SOD, CAT, and APX, indicating an inverse relationship. After treatments with Nano metal oxides, SOD, CAT, and APX represented higher activity as shown in **Fig 7B**.

Data in **Fig 7C** provides a visual illustration of the levels of various biochemical parameters under different nanoparticles treatments and the controls in *L. sativa* seedlings. Cluster 1 represents total protein content, total chlorophyll, and carotenoids, while Cluster 2 represents H_2_O_2_, MDA, SOD, CAT, and APX. In general, cluster 1 showed higher levels compared to cluster 2. Total protein content data obtained moderate to high levels across most treatments compared to the controls (infected plants and infected plants + Fungicide), exhibiting very high levels in the healthy plants. In addition, total chlorophyll and carotenoids showed a range of levels, generally showing moderate levels across many treatments but also including some high expressions in the healthy plants (C1) and SiO_2_ nanoparticles at 200 mg L^-1^. However, total protein contents, total chlorophyll, and carotenoids presented lower levels with CuO nanoparticles treatment at 200 mg L^-1^ in comparison to other treatments.

Also, carotenoids indicated the highest expressions in the healthy plants (C1) and γFe_2_O_3_ nanoparticles at 200 mg L^-1^. On the other hand, Cluster 2 of the heat map indicates that SOD, CAT, and APX represented higher levels after treatment with CuO and γFe_2_O_3_ nanoparticles at 200 mg L^-1^ compared to the other treatments and controls, while the lower levels were indicated in the healthy plants. Conversely, H_2_O_2_ and MDA presented high expressions through the treatments compared to the healthy plants (C1), but the highest expression was detected in the infected plants (C2). The differential expression and activity levels highlighted the impact of various treatments on these biochemical parameters.

### 3.7. Analysis of gene expression

**Fig 8:**
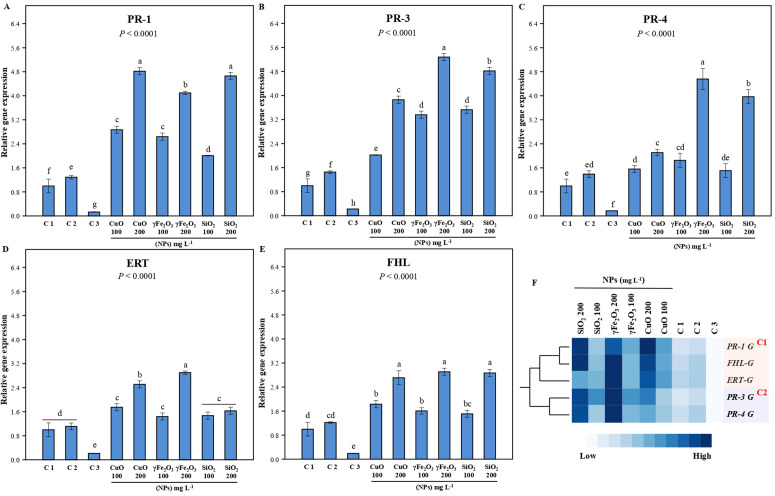
Changes in gene expression of all studied genes using qRT-PCR in the lettuce seedlings after treatment using three different nanoparticles, CuO, γFe_2_O_3_, and SiO_2_ at two different concentrations (100 and 200 mg L^-1^) on *L*. *sativa* infected seedlings. Healthy plants (C1), infected plants C2, and infected plants + Tolclofos-methyl (C3) were used as controls, (A) PR1, (B) PR3, (C) PR4, (D) ERT, and (E) FHL. *Actin* gene (accession number JX444700.1) was employed to calibrate relative expression (RQ). Data are presented as averages ± SEs of relative expression from 3 biological replicates for every cDNA sample. Values with different letters have significant differences at p < 0.05. (F) A two-way hierarchical cluster analysis and heat map illustrating the gene expression of *PR-1 G*, *FHL-G*, *ERT-G*, *PR-3 G*, and *PR-4 G* after treatments with CuO, γFe_2_O_3_, and SiO_2_ nanoparticles at different concentrations (100 and 200 mg L^-1^) compared to the controls (C1: healthy plants, C2: infected plants, C3: infected plants + Tolclofos-methyl) on lettuce (*Lactuca sativa* L.) seedlings. The similar responses to the nanoparticles were categorized into groups in clusters 1 and 2. Rows represent the expression of different genes, while the columns represent different treatments at different concentrations. The cells indicate the mean of the level of expression (n = 3).

The quantitative real-time PCR technique was utilized to precisely assess changes in the relative expression of examined genes. *Actin* gene (Ac: JX444700.1) was employed as an internal reference gene to calibrate the expression levels of the examined genes. The relative expression of *pathogenesis-related (PR) proteins*, such as *PR1 proteins* (antifungal), *PR3* (chitinases), *PR4 proteins* (antifungal), and *Ethylene-Responsive Transcription factor 1A gene (ERT1)* as a key regulatory in plant reaction to biotic stresses and the *fatty acid hydroperoxide lyase gene (FHL)* was investigated (**Fig 8**)*.* The results demonstrated that fungal infection induced a modest upregulation in the expression of all analyzed genes relative to the uninfected control plants. Conversely, fungicide treatment led to a significant downregulation in gene expression levels compared to both the untreated control and the group treated with nanoparticles.. All Nano metal oxides used in this study induced the transcription of all studied genes, with a significant increase in their expression as the concentration of Nano metal oxides increased. The highest transcription level of the *PR1 gene* was recorded at treatment with CuO at 200 mg L^-1^ and SiO at 200 mg L^-1^ treatments, about 4.8 and 4.6 folds respectively, followed by γFe_2_O_3_ at 200 mg L^-1^ with about 4.0 folds compared to untreated plants (**Fig 8A**). For the PR3 gene, the highest transcription level was detected with γFe_2_O_3_ at 200 mg L^-1^ (about ~ 5.2 fold) followed by SiO_2_ at 200 mg L^-1^ and CuO at 200 mg L^-1^ treatments (4.8 and 3.8 folds), respectively. Both treatments of γFe_2_O_3_ at 100 mg L^-1^ and SiO_2_ at 100 mg L^-1^ showed similar effects on the transcription level of the *PR3 gene* (**Fig 8B**).

The transcription level of the *PR4* gene was upregulated by about 4.5 folds with γFe_2_O_3_ at 200 mg L^-1^ treatment, followed by about 3.9 folds with SiO_2_ at 200 mg L^-1^ (**Fig 8C**). In contrast, all Nano metal oxide treatments with 100 mg L^-1^ concentration exhibited a non-significant increase in *PR4* gene expression in comparison with untreated plants. For the *ERT1* gene (**Fig 8D**), treatment with γFe_2_O_3_ at 200 mg L^-1^ recorded the highest transcription level, about 2.9-fold, followed by CuO at 200 mg L^-1^ with approximately 2.5-fold compared to the control. Other Nano metal oxide treatments exhibited the same effect on the transcription level of the *ERT1 gene*, with no significant differences among them. Generally, all nano metal oxides used in this investigation demonstrated a considerable increase in the gene expression of the *FHL* gene, with the highest expression level observed at the 200 concentration for all nano metal oxides (**Fig 8E**).

The heat map analysis was generated to illustrate a visual comparison of the gene expression levels across different nanoparticles treatments (CuO, γFe_2_O_3,_ and SiO_2_) at different concentrations and the controls in *L. sativa* seedlings, highlighting which genes are more expressed under certain treatments (**Fig 8F**). Two main clusters were detected; clusters 1 and 2 represented the expression of five genes, PR-1, FHL, ERT, PR-3, and PR-4. There was a clear difference between the expression of cluster 1 (PR-1, FHL, ERT) and cluster 2 (PR-3, PR-4). Cluster 1 genes generally indicated higher levels of expression, while cluster 2 genes were generally lower in expression with some variability across the treatments. CuO nanoparticles, γFe_2_O_3_ nanoparticles, and SiO_2_ nanoparticles at 200 mg L^-1^ showed very high levels of all gene expression, while at 100 mg L^-1,^ it exhibited moderate levels compared to the controls (low levels). Additionally, the lowest level of expression across all genes was detected in C3, the infected plants + Tolclofos-methyl treatment.

Plants have evolved various methods to protect themselves from diseases. These mechanisms rely primarily on *pathogenesis-related (PR) genes*, which become activated in response to pathogen attacks. These genes control the synthesis of different proteins, peptides, or substances that either kill pathogens or prevent pathogen infections [77]. PR-1 proteins are highly abundant in plant defensive responses, constituting around 2% of leaf protein in pathogen-infected tobacco [78]. The rise and presence of PR-1 proteins following infection, especially in the apoplast (the key interface in plant-microbe interactions), point to possible antibacterial activity. Numerous investigations have shown that the upregulation of *PR-1* in transgenic plants leads to enhanced resistance to fungi [79], indicating that *PR-1*’s broad antimicrobial action contributes to the observed enhanced resistance. Chitinases (*PR3* and *PR4*) are key enzymes involved in hydrolysis, found in many plants after infection with various pathogens. Chitin, a significant structural ingredient of the cell walls of numerous harmful fungi, is targeted by these enzymes. The quantity of chitinases significantly increased during infections, playing a critical part in the defense reaction against fungal infections by breaking down the cell walls [80]. The production of pathogenesis-related protein-like protein gene was elevated in a resistant genotype during *Alternaria solani* infection in tomato [81]. The results obtained in our study are consistent with previous research, which also reported an increase in the expression of the *PR-1, PR3,* and *PR4* genes in lettuce plants infected with *R. solani*. This finding further supports the notion of the important role of these genes against fungi, indicating that stimulating the expression of *PR-1, PR3,* and *PR4* genes in lettuce leads to increased resistance against *R. solani*. Importantly, increased expression of these genes was significantly correlated with a reduction in the severity of disease symptoms.

Nanoparticles serve a significant function in agricultural disease management, stimulating plant defense mechanisms [82]. When nanoparticles interact with plants, they can activate a variety of defense mechanisms, such as protective gene expression, oxidative signaling, and phytohormone production. These reactions allow plants to better recognize and battle invading pathogens, establishing an innate defense against diseases [83]. Moreover, this is consistent with several reports indicating that CuO nanoparticles employ various mechanisms to inhibit microbial pathogens [84]. In a related study, Lopez-Lima et al. [85] assessed the antifungal properties of copper nanoparticles at different concentrations against Fusarium oxysporum f. sp. lycopersici (FOL) under in vitro conditions. Similarly, El-Abeid et al. [86] demonstrated that CuO-Zs-nanoparticles exhibited enhanced antifungal activity in both laboratory and greenhouse trials, outperforming the commercial fungicide “Kocide 2000” and the biocontrol agent Trichoderma viride (1.5% WP) under both in vitro and in vivo conditions in controlling Fusarium root rot. Similarly, chitosan-coated ferrous nanoparticles induce the production of PR proteins and antioxidant enzyme-producing genes to combat bacterial leaf blight in rice [87]. Nanoparticles can induce the production of antimicrobial substances, such as *pathogenesis-related* (*PR*) proteins, hydrolytic enzymes, and phenolic compounds, which enhance plant defense [20,88]. These compounds inhibit pathogens, slowing their development and infection processes [89]. Furthermore, silica nanoparticles have been shown to enhance the production of antimicrobial hydrolytic enzymes, such as β-1,3-glucanase, pectinase, and chitinase, in tomato plants, thereby strengthening their defense against wilt-causing bacterial pathogens [90]. Our study also demonstrated that the nanomaterial oxide induced the expression of *PR1, PR3, and PR4 genes*, suggesting that CuO, γFe_2_O_3_, and SiO_2_ nanoparticles play a role in boosting the plant’s immune response against *R. solani*. This finding is further supported by the Disease Index results presented in **Fig 2**, which show that all nanoparticle oxides showed significant alleviated the symptoms of *R. solani* basal rot and resulted in lower AUDPC values. It is believed that the *PR1 gene* is linked to systemic acquired resistance, while the *PR3 and PR4 genes* contribute to the breakdown of the pathogen’s cell walls and possess antifungal properties. Additionally, the observed stimulation from the Nano material oxide treatment may be attributed to the activation of certain pathways and compounds, such as ROS, which in turn could trigger the plant’s defense mechanisms, including antioxidant enzymes.

Ethylene is necessary for several developmental stages and plays an important role in modulating plant abiotic and biotic stress responses. *ERF1* stimulates the transcription of defense-related genes that are dependent on ethylene and jasmonate. The *ERF1* gene plays a vital role in regulating ethylene/jasmonate-dependent defenses by integrating ethylene and jasmonate signals. Also, *LsERF1* regulated the ethylene and jasmonic pathways to provide resistance to necrotrophic fungi [91]. Analyzing the transcriptomes of Arabidopsis and lettuce plants infected with *B. cinerea* showed up-regulated expression levels of *LsERF1* at 18 and 24 hours after infection (hai), indicating that this transcription factor may be involved in providing resistance to this necrotrophic phytopathogen [92,93]. *LsERF1* in lettuce plants was significantly induced in the resistant genotype compared with the sensitive, with the greatest expression level indicating that its overexpression could be a crucial element in mediating resistance to *S. sclerotiorum* [94]. Numerous studies documented the notable overexpression of ethylene-related genes in plants treated with nanoparticles [95–97]. The nano-enabled activation of plant hormone signaling pathways, including jasmonic acid (JA), salicylic acid (SA), ethylene (ET), and abscisic acid (ABA), represents a novel strategy for enhancing plant immunity. This approach stimulates key defense-related pathways, thereby improving resistance to microbial pathogens [51]. Our results were in agreement with previous studies, demonstrating that treatment with nano-metal oxides, specifically γ-Fe₂O₃ and CuO at concentrations of 200 mg L ⁻ ¹, significantly upregulated the expression of the ERF1 gene in lettuce plants infected with *R. solani*. This increase in ERT1 expression correlated with a marked reduction in disease severity, as shown in **Fig 2**. Among the tested treatments, γ-Fe₂O₃ and CuO were the most effective in mitigating disease symptoms. These findings suggest a critical role for ERT1 in the lettuce defense response to *R. solani* and highlight the potential of γ-Fe₂O₃ and CuO nanoformulations as effective management strategies for controlling *R. solani* infections in both greenhouse and field conditions.

One important enzyme in the lipoxygenase system of plants is *hydroperoxide lyase (HPL)*. It can convert lipoxygenase-catalyzed fatty acid hydroperoxides into volatile aldehydes and oxoacids [98]. Several studies have revealed that *LsHPL1* plays an extensive function in modifying plant defense mechanisms, including the emission of green leaf volatiles (GLVs) and the control of genes linked with the jasmonic acid (JA) pathway [99]. Its products are specific scent components that can be used as food and perfume additives, as well as to keep insects and diseases away from plants [100]. It was determined that *HPL* influences the production of the jasmonic acid gene and the ejection of green leaf volatiles, which in turn controls the tomato’s defense response towards *Alternaria* and *Prodenia litura* [99]. Among the primary defense systems against fungus-related plant diseases can be anticipated to be HPL-derived catalysis of 13-hydroperoxides to C6-volatiles [101]. To author’s knowledge, this is the first detection of the role of CuO, γFe_2_O_3_, and SiO_2_ nanoparticles in the activation of the *fatty acid hydroperoxide lyase* (*FHL*) gene in lettuce plants’ response to *R. solani*. The activation of the *FHL* gene could be one of the multiple mechanisms of nano material oxide treatments to enhance plant defense responses, which contributes to the production of reactive aldehydes and plays a role in regulating the plant’s oxylipin pathways, which are crucial for defense against fungal pathogens like *R. solani*. The exact mechanisms underlying the nanoparticle-mediated activation of *FHL* remain an area of active research, but nanoparticles could potentially be harnessed as tools to improve plant immunity by modulating key defense pathways such as the oxylipin pathway and *FHL* expression.

Our findings suggest that the application of metal oxide nanoparticles (γ-Fe₂O₃, SiO₂, and CuO) may induce a controlled oxidative stress in plant tissues, initiating redox signaling pathways that activate defense-related gene expression. This includes the upregulation of *pathogenesis-related (PR) proteins* as well as *the Ethylene-Responsive Transcription Factor 1A gene (ERT1)*, *the fatty acid hydroperoxide lyase (FHL) gene,* and an antioxidant, which collectively contribute to enhanced redox homeostasis and strengthened defense responses against Rhizoctonia solani. In addition, the nanoparticles may function as elicitors by mimicking pathogen-associated molecular patterns, thereby engaging pattern recognition receptors and priming the plant immune system. This priming potentially facilitates a faster and more effective defense response upon pathogen exposure. Together, these molecular responses are consistent with the observed upregulation of resistance-associated genes and the corresponding reduction in root rot symptoms, indicating a potential role of nanoparticle treatment in enhancing disease resistance in lettuce.

### 3.8. Residual Si, Cu, and Fe concentrations in Lettuce seedlings

Silicon, Copper, and Iron concentrations in lettuce seedlings after being treated with nanoparticles and their control were listed in **Table 2**. Whole plants, including roots and shoots, were used to measure the metal concentrations in plant tissue. The plant’s average Si concentrations were 9.52 ± 0.1 and 10.44 ± 0.04 mg kg^-1^ DW (dry weight) for 100 and 200 mg L^-1^, respectively, in comparison to the control treatment. Copper concentration in lettuce plant recorded its maximum residual concentration when the plant was treated with 200 mg L^-1^ CuO (109.72 ± 4.2 mg kg^-1^ DW), followed by 100 mg L^-1^ CuO (70.47 ± 2.5 mg kg-1 DW). The data demonstrate that the level of copper in plants increased with increasing CuO concentrations. The same trend was obtained with γFe_2_O_3_ treatment, with Fe concentrations of 58.38 ± 1.3 and 109.58 ± 4.6 mg kg^-1^Dw for 100 mg L^-1^ and 200 mg L^-1^, respectively, as shown in **Table 2**.

**Table 2:** Silicon, Copper, and Iron composition (mg kg^- 1^) of lettuce (*Lactuca sativa L.*) plants at the end of the experiment.

Treatment		Si	Cu	Fe
C1		8.55 ± 0.05^bc^	10.96 ± 0.3^c^	27.14 ± 1.2^c^
C2		5.85 ± 0.2^e^	3.92 ± 0.2^c^	7.34 ± 0.4^f^
C3		7.93 ± 0.1 cd	8.37 ± 0.3^c^	21.91 ± 1.3 cd
CuO nanoparticles	100 mg L^ − 1^	7.57 ± 0.1 cd	**70.47 ± 2.5** ^**b**^	12.91 ± 0.1^def^
	200 mg L^ − 1^	7.33 ± 0.1^d^	**109.72 ± 4.2** ^**a**^	12.38 ± 0.9^ef^
γFe_2_O_3_ nanoparticles	100 mg L^-1^	7.96 ± 0.1 cd	9.18 ± 0.2^c^	**58.38 ± 1.3** ^**b**^
	200mg L^-1^	7.96 ± 0.05 cd	8.36 ± 0.3^c^	**109.58 ± 4.6** ^**a**^
SiO_2_ nanoparticles	100 mg L^ − 1^	**9.52 ± 0.1** ^**ab**^	8.51 ± 0.1^c^	18.63 ± 0.5^cde^
	200 mg L^ − 1^	**10.44 ± 0.04** ^**a**^	9.29 ± 0.05^c^	22.25 ± 0.8^cde^

*Where*: C1: Healthy plants, C2: Infected plants, C3: Infected plants + Tolclofos-methyl (Fungicide)

It is worth noting that treatment with 200 mg L^-1^ CuO caused a significantly decreased Fe concentration in plant tissues in comparison with the control. No significant effect was obtained for silicon on either Cu or Fe concentrations.

All plants require micronutrients like Cu, Fe, Zn, and Mn, which are implicated in several metabolic processes. While the majority of plant species do not require silicon (Si), plants that contain Si may respond differently to mineral-deficient stress or mineral toxicity [102,103]. For example, introducing Si to cucumber plants under Cu stress enhances the build-up of components (proteins and organic acids) that bind Cu and may bind excess Cu, reducing the level of oxidative stress [104].

A recent study on lettuce plants examined the harmful effects of high levels of copper (Cu) on the roots [105,106]. Another study found that 100 mg L^−1^ of CuO nanoparticles generated toxicity in lettuce plants, while 100 mg L^−1^ of γFe_2_O_3_ or SiO_2_ nanoparticles positively influenced root diameter, tissue structure, and numerous anatomical parameters [23]. Cu stress led to nutritional deficiencies in plant leaves [107]. Examined and contrasted the effects of synthesized CuO nanoparticles with bulk Cu on the growth parameters of wheat. They concluded that both forms of Cu inputs (nanoparticles and bulk) considerably impede root development compared to the control. Bulk Cu and CuO nanoparticles had corresponding effective toxic doses (EC_50_) of 0.37 mg L^-1^ and 0.94 mg L^-1^, respectively. The findings indicated that due to the reduced dissolution of CuO nanoparticles, approximately 2.5 times the concentration of CuO nanoparticles is equivalent to the toxic dose of bulk copper [108]. Our findings indicate that Cu phytotoxicity has a nanoscale effect, emphasizing the importance of assessing CuO nanoparticles dissolution near lettuce plant roots under realistic environmental conditions to understand plant-induced changes. Cu-nanoparticles are frequently claimed to have lower short-term toxicity and higher long-term effectiveness against fungus disease than their bulk and ionic equivalents [23,109–111]. The obtained results showed that a significant amount of Fe was accumulated in lettuce plants. Generally, in terms of total chlorophyll content, γFe_2_O_3_ nanoparticles may significantly boost lettuce growth. The evidence of decreased antioxidant enzyme activities indicated that the addition of γFe_2_O_3_ did not cause peroxidation in the plants; rather, they induced growth by generating specific levels of ROS, which are signaling molecules that cause the elongation of roots and the development of plants.

Due to their nano-effects, nanoparticles can penetrate plant cells, unlike bulk nanoparticles (in micrometers), and accumulate in plant tissues [71]. Peanut plants treated with EDTA-Fe and Fe_2_O_3_ nanoparticles had higher Fe levels than those in the control group [71]. On the other hand, the Fe_2_O_3_ nanoparticles absorbed into the sandy soil and increased the plant’s availability of Fe. These findings demonstrate that Fe_2_O_3_ nanoparticles can be used instead of conventional Fe fertilizers while growing peanut plants. In another study, the transport and the uptake of Fe_2_O_3_ nanoparticles in watermelon plants were examined [112]. This research reported that watermelon plants can absorb a sizable amount of Fe_2_O_3_ nanoparticles floating in a liquid medium and translocate them throughout the plant tissues.

## 4. Conclusions

This study aimed to determine the induced resistance mechanism against *R*. *solani* by applying some metal oxide nanoparticles, such as SiO_2_, CuO, and γFe_2_O_3_ nanoparticles, to lettuce plants. Also, investigate their effects on some physiological characteristics of lettuce plants. Based on the obtained results, applying SiO_2_, CuO, and γFe_2_O_3_ nanoparticles to lettuce plants effectively reduced the symptoms of *R. solani* basal rot. Additionally, the use of nanoparticles, particularly 100 mg L^−1^ concentration of nanoparticles, enhanced certain growth characteristics in lettuce plants. Furthermore, all nanoparticles in our study activated the lettuce defense system by raising SOD, CAT, and APX activity and induced an increase in the transcription level of all studied genes, which addresses the role of applied treatment in the regulation of the expression of pathogenesis-related genes. The obtained result revealed the activation of the *fatty acid hydroperoxide lyase* (*FHL*) gene in lettuce plants treated with CuO, γFe_2_O_3_, and SiO_2_ nanoparticles in response to *R. solani* as a new approach of Nanoparticle mode of action in the acquisition of resistance. Treatment with γFe_2_O_3_ nanoparticles was the best compared to other nanoparticles. As a result, these treatments might be recommended as a promising option for alleviating the fatal effects of *R. solani* on lettuce plants in both greenhouses and open fields by inducing systemic resistance of lettuce plants.

Despite promising greenhouse results, field trials are needed to confirm the effectiveness of metal oxide nanoparticles under variable environmental conditions. Key research gaps include understanding their long-term impact on soil health, plant metabolism, and food safety. Further studies should assess nanoparticle persistence, degradation, and potential ecological risks to ensure safe and sustainable agricultural use.

## Supporting information

S1 Fig Lettuce plants exhibiting root rot symptoms under different treatments: γFe₂O₃, CuO, SiO₂ nanoparticles, C3 (infected plants treated with Tolclofos-methyl), and C2 (infected untreated plants).
(JPG)

## Abbreviations

*R Solani,*: *Rhizoctonia Solani*
SiO_2_: Silicon dioxide
CuO: Copper oxide
γFe_2_O_3_: Gamma iron oxide
SR: Systemic resistance
H_2_O_2_: Hydrogen peroxide
MDA: Malondialdehyde
AUDPC: Area Under Disease Progression Curve
SOD: Superoxide dismutase
CAT: Catalase
APX: Ascorbate Peroxidase
qRT-PCR: Real-time polymerase chain reaction
PR1, PR3, PR4: pathogenesis-related protein genes
ERT1: Ethylene-Responsive Transcription Factor 1A gene
FHL: The fatty acid hydroperoxide lyase gene
RQ: Relative expression
SEM: Scanning electron microscope.
