# Supplementary figures and images for: Stimulation of resistance genes and antioxidant enzymes in lettuce by nano metal oxides against root rot caused by Rhizoctonia solani
Source: PLoS One. 2025 Oct 14;20(10):e0334506. doi: 10.1371/journal.pone.0334506 (PMC12520340; doi:10.1371/journal.pone.0334506)

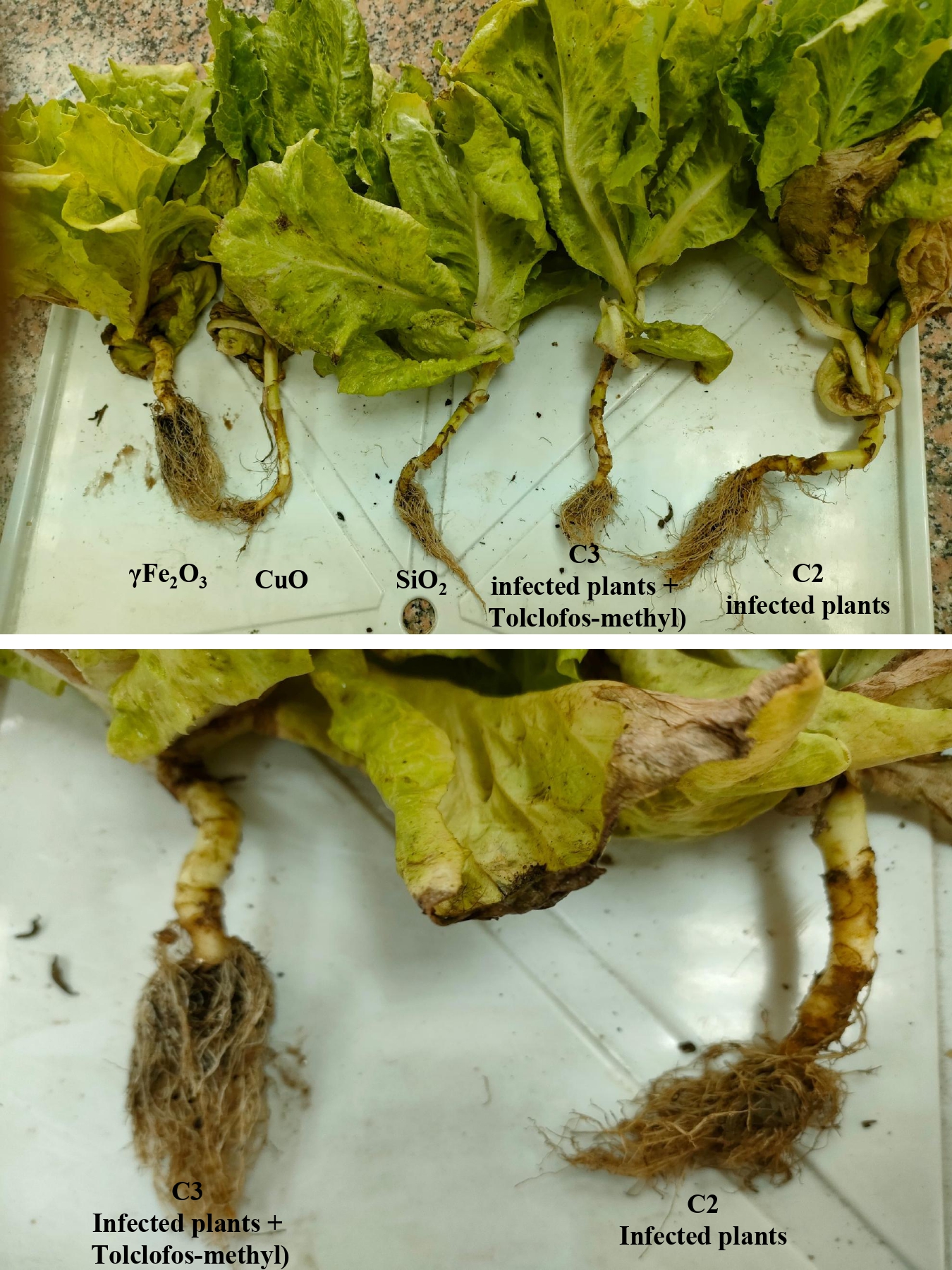

Supplement: S1 Fig — Lettuce plants exhibiting root rot symptoms under different treatments: γFe₂O₃, CuO, SiO₂ nanoparticles, C3 (infected plants treated with Tolclofos-methyl), and C2 (infected untreated plants). (JPG) [file pone.0334506.s001.jpg]
